# Supplementary material for: Reciprocal Regulation of Shh Trafficking and H2O2 Levels via a Noncanonical BOC-Rac1 Pathway
Source: Antioxidants (Basel). 2022 Apr 5;11(4):718. doi: 10.3390/antiox11040718 (PMC9025708; doi:10.3390/antiox11040718)
Supplement: Supplementary file 1 [file antioxidants-11-00718-s001.zip › antioxidants-1618403-supplementary.pdf]

Supplementary information

Reciprocal regulation of Shh trafficking and H_2O_2 levels via a noncanonical BOC-Rac1 pathway

Marion Thauvin, Irène Amblard, Christine Rampon, Aurélien Mourton, Isabelle Queguiner, Chenge Li, Arnaud Gautier, Alain Joliot, Michel Volovitch, Sophie Vriz

Contents

Figure S1-S2
Tables S1-S4

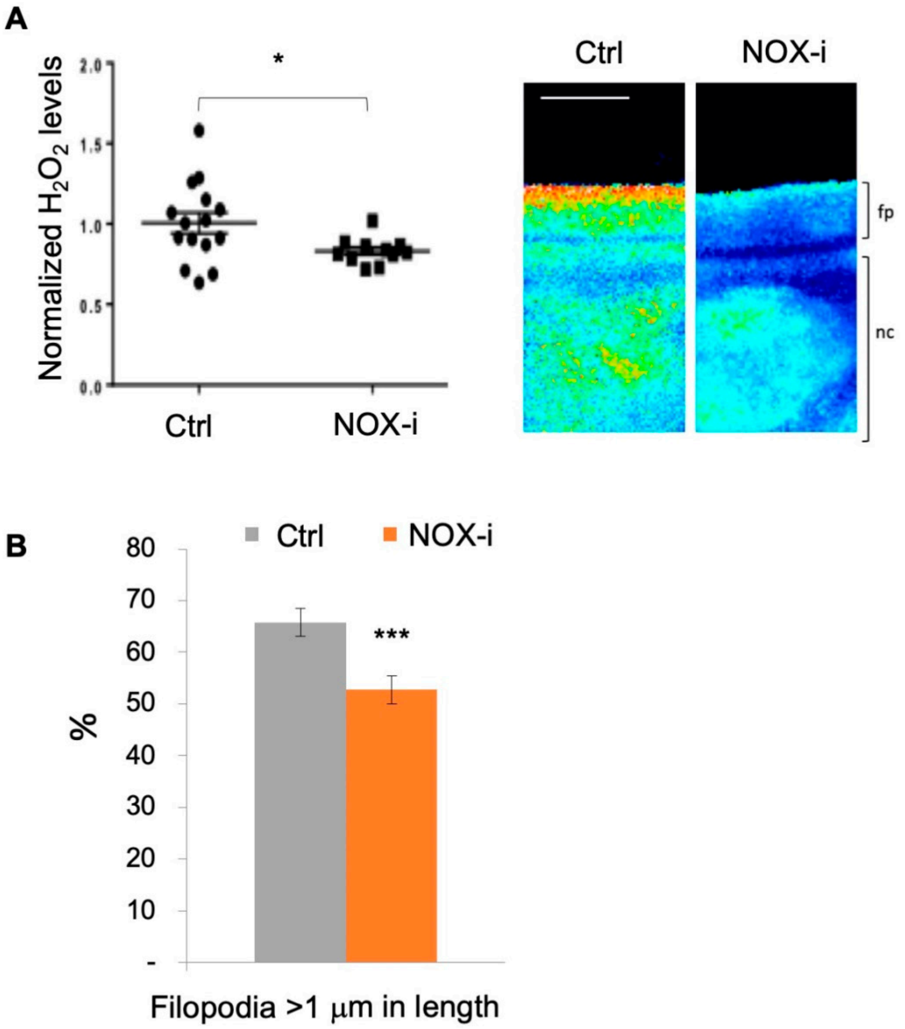

Figure S1. NOX-i treatment induces a reduction of H_2O_2 levels as well as a reduction of filopodia length in the MFP.

A, H_2O_2 levels decreased after NOX-i treatment. 2.4Shha-ABC:GalA-FF/UAS:HyPer7 embryos were incubated in NOX-i (10 µM), and H_2O_2 levels were quantified in the MFP at 46 hpf. Representative images are shown. fp: floor plate; nc: notochord. Scale Bar: 10 µm.

B, Quantification of filopodia length in MFP cells (see Methods) in Ctrl and NOX-I treated larvae. Details on statistics in Material and Methods

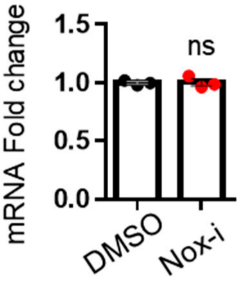

Figure S2. ***Shha*** **expression is not affected by Nox-i treatment**
Gene expression was analyzed by quantitative RT–PCR after Nox-i or DMSO treatment. The level in the DMSO-treated sample was set to 1. The error bars indicate the SEM values.

Table S1: Plasmids used in this study

Plasmid number	Plasmid name	Regulatory sequences	Expressed protein	purpose	ref
65	pCS2transposase	SP6	Tol2 transposase	transgenesis	[53]
1070	pT22i2Shh24Gal4F	2.4Shha-ABC	Gal4BD-FF	transgenesis	this paper
1190	pT22i5uasHyper7	5xUAS	HyPer7	transgenesis	this paper
1242	pT22i5uasm4C5DaoChe	5xUAS	Igk-mb5-DAO-mCherry	transgenesis	this paper
1243	pT22i5uasm4C5CatChe	5xUAS	Igk-mb5-CAT-mCherry	transgenesis	this paper
1261	pT2i5biGfpF•Shh-SbpChe	5xUAS bidirectional	(i) GFP-Farn<br>(ii) Shh-SBP-mCherry	transgenesis	this paper
237	pCSmChF	SP6	mCherry-Farn	mRNA synthesis	this paper
1313	pcDNA7LGBi	CMV-tetOx2	LgBiT	stable cell line	[43]
1314	pcDNA2LsiLGBiCD5	CMV-tetOx2	IL2-LgBiT-mb5	stable cell line	this paper
754	pcDNA7Hyper1	CMV-tetOx2	HyPer1	stable cell line	[23]
1036	pcDNA2LckHyper	CMV-tetOx2	Lck-HyPer1	stable cell line	this paper
911	pT2iC6mShhYfaChe	sCMV	Shh-YFast-mCherry	transient *ex vivo* expr	this paper
669	pT2iC6mShhChe	sCMV	Shh-mCherry	transient *ex vivo* expr	this paper
1330	pKhCTagScLgBiT	T7	CTagSc-LgBiT	bac prod	this paper
832	pStrKdel_ShhSbpChe	CMV	sti-STRP-kdel-ires-Shh-SBP-mCherry	transient *ex vivo* expr	this paper
1265	pCL9StKDEL	CMV	sti-STRP-kdel	transient *ex vivo* expr	this paper
1237	pcDNA2SbpShhSbi	CMV-tetOx2	Shh-SBP-HiBiT	transient *ex vivo* expr	this paper
1311	pcDNA2SilGfpSbpSbi	CMV-tetOx2	IL2-GFP-SBP-HiBiT	transient *ex vivo* expr	this paper
1312	pcDNA2silSbiChe	CMV-tetOx2	IL2-HiBiT-mCherry	transient *ex vivo* expr	this paper
966	pcDNA2LckCheDao	CMV-tetOx2	Lck-mCherry-DAO	transient *ex vivo* expr	this paper
669	pT2iC6mShhChe	sCMV	Shh-mCherry	transient *ex vivo* expr	this paper

CAT: catalase deprived from its lysosome-targeting signal; CTagSC: His6-bacterial CherryTag-Scission protease site for bacterial expression; DAO: D-Aminoacid oxidase; Farn: C-terminal farnesylation+palmitoylation signal from Ha-Ras; Gal4BD-FF: Gal4 DNA binding domain fused to dimerized minimal activation domain F; HiBiT: small fragment of split nanoluciferase; HyPer: H_2O_2 ratiometric probe; Igk: signal peptide from kappa light chain; IL2: signal peptide from interleukin2; IRES: Internal Ribosome Entry Site; kdel: classical ER retrieval signal; Lck: N-terminal myristoylation+palmitoylation signal from Lck tyrosine kinase; mb5 : minimal transmembrane domain form CD4; LgBiT: large fragment of split nanoluciferase; SBP: streptavidin binding peptide; Shh; Sonic Hedgehog; sti: signal peptide from stromal interaction molecule 1 STIM1; STRP: core streptavidin; YFAST: Yellow fluorogen-activated peptide

Table S2: Stable HeLa cell lines used in this study

**Name**	**Construct used for HeLa Flp-In**	**Expressed protein**	**ref**
GBi	pcDNA7LGBi	LgBiT	this study
siLGBimb5	pcDNA4siLGBiCD5	IL2-LgBiT-mb5	this study
LckHyPer	pcDNA7LckHyPer	Lck-HyPer1	this study
HyPer	pcDNA7Hyper	HyPer1	[23]

Table S3. Sample sizes and number of replicates.

Figure	condition	Sample size	Mean	Std. Deviation	Std. Error of Mean	P-value	Confidence interval
**Figure 2**							
2C	Shh	4					
	SecGFP	4					
2E	Shh t=5min	2	16	1.697	1.2	T=5min<br>Shh vs SecmCh<br>P>0.9999<br>t=30min<br>Shh vs SecmCh<br>P<0.0001<br><br>t=60min<br>Shh vs SecmCh<br>P<0.0001<br><br>t=75min<br>Shh vs SecmCh<br>P=0.0491	
	Shh t=30min	8	100	23.11	8.17		
	Shh t=60min	5	104.2	16.62	7.432		
	Shh t=75min	2	112	5.657	4		
	Secm Ch t=5min	2	11.5	7.778	5.5		
	SecmCh t=30min	7	18.43	16.22	6.129		
	SecmCh t=60min	4	36	7.348	3.674		
	SecmCh t=75min	2	25.5	27.58	19.5		
2G	Ctrl (A)	11	0.1	0.02246	0.006772	A vs B p>0.9999<br>A vs C p=0.7699<br>A vs D p=0.0919<br>A vs E p<0.0001<br>A vs F p<0.0001	
	1h (B)	10	0.1017	0.03336	0.01055		
	2h (C)	6	0.1387	0.0549	0.02241		
	3h (D)	8	0.1726	0.05061	0.01789		
	4h (E)	7	0.2578	0.07452	0.02816		
	4h30 (F)	8	0.2518	0.09564	0.03381		
	Nox-i	11	0.83	0,08184	0,02467		

**Figure 3B**							
Shh	Ctrl	14	100	25.46	6.804	0.0007	
	D-Ala	13	53.96	25.69	7.126		
SecGFP	Ctrl	14	100	30.19	8.069	0.9966	
	D-Ala	12	101.7	22.06	6.368		
**Figure 3C**							
Shh	Ctrl	15	100	16.06	4.147	<0.0001	
	CAT	13	155.5	23.20	6.434		
SecGFP	Ctrl	8	100	9.794	3.463	0.0173	
	CAT	7	75.71	21.90	8.277		
**Figure 3E**							
Shh	Ctrl	8	100	24.91	8.808	0.0048	
	D-Ala	8	144.6	39.1	13.83		
SecCh	Ctrl	8	7.579	21.88	7.736	0.9380	
	D-Ala	7	12	11.45	4.326		
**Figure 3F**							
Shh	Ctrl	6	100	19.18	7.832	0.0007	
	CAT	6	52.46	8.507	3.473		
SecCh	Ctrl	5	32.7	23.72	10.61	0.9341	
	CAT	5	28.78	21.23	9.493		
**Figure 3H**							

	Ctrl t=0 (A)	10	0,1	0.0255	0.008065	A vs B p=0.0091<br>B vs C p<0.0001	
	Ctrl t=4h (B)	9	0.1395	0.02436	0.008119		
	D-Ala t=4h (C)	11	0.2162	0.03876	0.01169		
**Figure 3I**							
	Ctrl t=0 (A)	6	0,1	0.03096	0.01264	A vs B p<0.0001<br>B vs C p=0.0066	
	Ctrl t=4h (B)	6	0.2705	0.07288	0.02975		
	CAT t=4h (C)	6	0.1697	0.02609	0.01065		

**Figure 4B**							
	26 hpf	8	1,125	0,08582	0,03034	<0,0001	0,95
	28 hpf	8	1,053	0,07437	0,02629	<0,0001	0,95
	30 hpf	13	1,000	0,07127	0,01977		0,95
	33 hpf	5	0,9074	0,07615	0,03406	<0,0001	0,95
	39 hpf	5	0,8569	0,04880	0,02182	0,0005	0,95
	45 hpf	5	0,8545	0,03733	0,01669	0,0027	0,95
**Figure 4D**							
	25 hpf	12					
	29 hpf	12					
	31 hpf	24					
	35 hpf	12					
	45 hpf	11					

**Figure 5C**							
	Ctrl ≤9	29	58.3%	10.06			
	Ctrl ≥10	29	41.7%	10.06			
	D-Ala	24	31%	8.59		<0.0001	0.95
	D-Ala	24	69%	8.59		<0.0001	0.95
**Figure 5D**							
	Ctrl	243	20%	2.57		<0.0001	0.95
	D-Ala	302	24%	2.46			
**Figure 5F**							
	Ctrl	15	6.2	3.14	0.81		
	Nox-i	22	12.05	5.62	1.2	<0.01	0,95
	HH-i	5	0	0	0	<0.05	0,95

**Figure 6A**							
mCherry	Exp- (A)	50	100	32.76	4.634	A vs B p=0.5451<br>C vs D p=0.3243<br>A vs C p=0.0392<br>B vs D p<0.0001	
	Exp+ (B)	15	88.43	34.2	8.830		
Shh-mCherry	Exp- (C)	14	133.3	29.17	7.797		
	Exp+ (D)	43	150	50.69	7.716		
**Figure 6D**							
	Ctrl	82	100	41.8	4.616	<0.0001	
	Shh	104	141.4	42.63	4.181		
**Figure 6E**							

	Ctrl	40	923.9	806.9	127.6	<0.0001	
	Shh	34	1940	1219	209.1		
**Figure 6F**							
	Ctrl (A)	113	100	26.1	2.456	A vs B p<0.0001<br>C vs D p<0.0001<br>E vs F p=0.8904	
	Shh (B)	128	143.7	48.08	4.250		
	Shh-i (C)	50	95.57	31.02	4.386		
	Shh+Shhi (D)	46	138.9	57.57	8.488		
	NOX-i (E)	58	76.87	29.65	3.894		
	Shh+NOX-i (F)	54	81.45	16.33	2.222		
**Figure 6G**							
	Ctrl (A)	111	100	29.47	2.798	A vs B p<0.0001<br>C vs D p=0.3685<br>E vs F p=0.9802<br>G vs H p=0.9308	
	Shh (B)	126	180	51.64	4.601		
	Rac1-i (C)	57	106.7	30.53	4.044		
	Shh+Rac1-i (D)	51	119.5	31.6	4.424		
	DOCK-i (E)	33	122.9	43.48	7.57		
	Shh+DOCK-i (F)	52	127.4	44.69	6.198		
	Rac1-i+DOCK-i (G)	25	110.3	28.44	5.688		
	Shh+ Rac1-i+DOCK-I (H)	22	118.7	41.37	8.821		

**Figure Sup1A**							
	Ctrl	14	1.0	0,2506	0,06472	0,030	0,95
	NOX-i	11	0.83	0,08184	0,02467		

**Figure Sup1B**							
	Ctrl	298	65.77	2.75	0.1592	<0.0001	0.95
	NOX-i	334	52.69	2.73	0.1495		

**Figure Sup2**	condition	Sample size	Mean	Std. Deviation	Std. Error of Mean	P-value
	DMSO	3	1.000	0.02160	0.05068	0.9146
	HH-i	3	1.004	0.005068	0.02926	

Table S4. Shh pathway member expression in cell lines

A: data from The Human Protein Atlas/Cell line database (https://www.proteinatlas.org/humanproteome/cell+line).
nTPM: number of transcripts per million (mRNA are considered expressed for mTPM>1).
B: this paper, determined by quantitative RT-PCR (see Methods)
nd : non detected, un : untested. Positive value indicates that gene of interest is x fold more expressed than gapdh and negative values x fold less than gapdh

**A**	HeLa nTPM
shh	0
ihh	0
dhh	0
smo	0
ptch1	3.0
ptch2	0.6
cdon	6.5
elmo2	18.7
elmo3	4.8
dock1	18.8
dock2	3.4
dock3	1.2
dock4	5.8
dock5	40.5

**B**	HeLa ΔCt	HEK	Universal probe ID	Left primer	Right primer
gadph			68	ccc cgg ttt cta taa att gag c	ctt ccc cat ggt gtc tga g
hhat	12.6±2.2	un	22	gct ggg agt cac tgt gga g	gct tgt ggg gag aag tat cg
disp1	7.0±1.4	un	43	tgt gca atg tag ata att cca gga t	gcg atg tag ttt ccc agt gtc
ptch1	3.8±0.2	un	17	tgg gat taa aag cag cga ac	cga ctt act cgt cct cca act t
ptch2	-4.8±1.1	un	17	tgg aac agc tct ggg tag aag	ccc agc ttc tcc ttg gtg ta
scube2	-45±9.3	un	17	agc tgc cat cca cag tac aa	cca ctg atg tgg tgt tgc tc
smo	nd	-3.2	22	gct tcc ggg act atg tgc ta	aaa cat ggc aaa cag gtt ga
boc	3.3±4.6	un	6	cgc caa ccc tct act atg tgg t	aat gcc aga gat ggt cca at

nox1	nd	un	43	ccc agc aga agg ttg tga tt	ttg agg ggc aat taa caa aga
nox2	-5.2±0.5	un	6	aca att gca agt caa cac cct a	ctc agg gtt tca gcc aag g
nox3	nd	un	22	tca agt cca ggc att gtg tt	aac agg ccg tca cag gaa
nox4	nd	un	22	aac caa ggg cca gag tat ca	gct gag gct ctg ctt aga cac
